# Unique Bioactives from Zombie Fungus (*Cordyceps*) as Promising Multitargeted Neuroprotective Agents

**DOI:** 10.3390/nu16010102

**Published:** 2023-12-27

**Authors:** Himadri Sharma, Niti Sharma, Seong Soo A. An

**Affiliations:** Department of Bionano Technology, Gachon Bionano Research Institute, Gachon University, 1342 Seongnam-daero, Sujeong-gu, Seongnam-si 461-701, Gyeonggi-do, Republic of Korea; himadri@gachon.ac.kr

**Keywords:** *Cordyceps*, zombie fungus, neuroprotection, neuroinflammation, oxidative stress, neurodegenerative diseases, neurotrauma

## Abstract

*Cordyceps*, also known as “zombie fungus”, is a non-poisonous mushroom that parasitizes insects for growth and development by manipulating the host system in a way that makes the victim behave like a “zombie”. These species produce promising bioactive metabolites, like adenosine, β-glucans, cordycepin, and ergosterol. *Cordyceps* has been used in traditional medicine due to its immense health benefits, as it boosts stamina, appetite, immunity, longevity, libido, memory, and sleep. Neuronal loss is the typical feature of neurodegenerative diseases (NDs) (Alzheimer’s disease (AD), Parkinson’s disease (PD), multiple sclerosis (MS), amyotrophic lateral sclerosis (ALS)) and neurotrauma. Both these conditions share common pathophysiological features, like oxidative stress, neuroinflammation, and glutamatergic excitotoxicity. *Cordyceps* bioactives (adenosine, N^6^-(2-hydroxyethyl)-adenosine, ergosta-7, 9 (11), 22-trien-3β-ol, active peptides, and polysaccharides) exert potential antioxidant, anti-inflammatory, and anti-apoptotic activities and display beneficial effects in the management and/or treatment of neurodegenerative disorders in vitro and in vivo. Although a considerable list of compounds is available from *Cordyceps*, only a few have been evaluated for their neuroprotective potential and still lack information for clinical trials. In this review, the neuroprotective mechanisms and safety profile of *Cordyceps* extracts/bioactives have been discussed, which might be helpful in the identification of novel potential therapeutic entities in the future.

## 1. Introduction

Neurodegenerative diseases (NDs) result from gradual loss of neuronal function, which ultimately causes cell death. These diseases remain incurable because of the complexity of brain function, leading to devastating neurological disorders. An increase in the incidence rate of NDs has been observed owing to rising life expectancy [1]. An imbalance between antioxidant and pro-oxidant species causes increased oxidative stress [2]. Increased reactive oxygen species (ROS)/reactive nitrogen species (RNS) in cells and tissues interfere with cellular mechanisms, such as mitochondrial dysfunction, leading to a decrease in energy production. Increased oxidative stress may lead to neuronal cell loss, a common feature of various NDs [2,3,4]. Oxidative stress, along with the production of cytokines, chemokines, and other secondary messengers, is the underlying cause of neuroinflammation. The expression levels of inflammatory cytokines positively correlate with the levels of neurotrophic factors [5]. Regulated inflammatory processes are important for maintaining tissue homeostasis and proper functioning, and extensive inflammation can lead to additional cell injury [6]. Neuroinflammation can be considered a double-edged sword, as it may cause damaging effects in NDs [7], while minor inflammation may be beneficial for recovery under some conditions [6,8,9,10]. Certain inflammatory inducers produce neurotoxic substances that amplify disease symptoms. These neurotoxic factors are associated with several NDs, like Alzheimer’s disease (AD), Parkinson’s disease (PD), multiple sclerosis (MS), and amyotrophic lateral sclerosis (ALS) [11,12]. Neuroinflammation is the major cause of ischemic stroke. M1-type microglial cells play a major role in prolonged inflammation, leading to brain tissue damage [13]. Although inflammation may not always be the initiating factor of these diseases, other sources of neuroinflammation include brain trauma, injury, and infection. Mitochondrial dysfunction, gliosis, and accumulation of abnormal protein aggregates also play critical roles in neurodegeneration [14]. Advanced glycation end products (AGEs) are formed by a non-enzymatic reaction (Maillard reaction) between the carbonyl group of reducing sugars and the amino group of amino acids. AGEs interact with the receptors of advanced glycation end products (RAGEs), initiating the activation of several pathways, including oxidative stress, and inflammation [15]. AGEs are also generated in food during thermal processing, like the pasteurization of dairy products [16,17] and immunomodulating gut bacteria. N^ε^-(carboxymethyl)lysine (CML) is an AGE that interacts with RAGEs and releases inflammatory cytokines, ultimately increasing oxidative stress [17]. Small-molecule therapeutics that interfere with AGE–RAGE interactions inhibit the inflammatory cascade and attenuate disease development [18]. Researchers worldwide have attempted to identify an effective cure for NDs. Despite several attempts to identify drugs that can reduce the symptoms of neurodegeneration, no permanent cure has been found. Hence, there is a critical need to identify bioactive compounds in nature and investigate their effects on neurodegeneration associated with NDs or neurotrauma.

*Cordyceps*, commonly known as the “caterpillar fungi”, “zombie fungus”, “Viagra of the Himalayas”, and “*Yartsa gunbu*”, belongs to the phylum Ascomycetes (Sac fungi). *Cordyceps* encompasses approximately 750 species that are distributed in different parts of the world, especially in temperate regions at altitudes over 3800 m. *Cordyceps* is the most expensive mushroom, with some species costing approximately USD 20,000/kg [19]. All *Cordyceps* species survive by invading their hosts with selective specificity. Most of these species parasitize insects and other arthropods, whereas a few invade *Elaphomyces* (Truffle genus) [20]. In brief, the life cycle of *Cordyceps* is initiated by spore germination, followed by the growth of hyphae by absorbing nutrients from the soil, invading the host, penetrating the host’s exoskeleton, and surviving in its tissues. After the host’s death, the fungus continues to grow, fruiting bodies sprout from the host’s head and release spores, and the cycle continues [21]. As this fungus manipulates the host system, the victim behaves like a “zombie”, with frequent convulsions; it is also called “zombie fungus” [22]. Due to these typical growth and survival tactics, these species synthesize promising bioactive metabolites, like anthraquinones, pyridines, cytochalasin, cyclic peptides, bioxanthracenes, polyketide, dihydrobenzofurans, glycans (β-glucans), alkaloids, phenols, flavonoids, terpenes, sterols (ergosterol), naphthoquinones, and nucleosides (adenosine, cordycepin) with broad therapeutic applications [23,24,25]. *Cordyceps* has been used in traditional Chinese medicine (TCM) health tonics [26] and for its medicinal properties, such as anticancer [27,28,29], antihyperglycemic [30,31], antifatigue [32], hepatoprotective [33], spermatogenic [34], hypolipidemic [35,36], antihypertensive [37,38], anti-inflammatory [39,40], nephroregulatory [41,42,43], antifibrosis [44], and immunomodulatory [45,46] properties. Although more than 750 *Cordyceps* species have been identified, only a few (*C. militaris*, *C. ophioglossoides*, *C. sinensis*, *C. cicadae*) have been studied for their neuroprotective activities.

Previous reviews of *Cordyceps* have focused on their nomenclature, structural elucidation, traditional use, and nutraceutical and pharmacological activities [23,34,46,47,48,49,50,51,52,53,54,55,56]. A bioactive compound (cordycepin) from this fungus has been a compound of choice among researchers, and most neuroprotective studies have been conducted using this compound, while information on other compounds is scarce. Hence, we present a comprehensive review of the neuroprotective mechanism and safety profile of the extracts and the active metabolites from *Cordyceps* investigated so far on neurotrauma and NDs.

## 2. Methods

This study presents a comprehensive review of the existing published scientific works from various databases (PubMed, Google Scholar, and Science Direct) until August 2023 on the key bioactive metabolites and neuroprotective potential of various species of *Cordyceps* to support its use in the treatment of searing diseases. The search terms used were “cordyceps” or “cordyceps bioactive” or “cordyceps metabolites” with the filter “neuroprotection”, “neurotrauma”, “neurodegenerative diseases”, “in vivo”, “in vitro” and “English”. Studies relevant to chemical analyses, medicinal applications, safety, toxicity, and neurodegeneration were selected.

## 3. Neuroprotective Potential of *Cordyceps* Extracts

Of all *Cordyceps* species, only 35 have been characterized [23], of which *C. militaris* and *C. sinensis* are the two most widely studied. *C. sinensis* is a rare and expensive species that is difficult to cultivate, whereas *C. militaris* is a successful commercially grown species and is considered an alternative to *C. sinensis* [57]. By changing the culture conditions, the concentrations of bioactive compounds can be manipulated.

Several extraction methods and solvents have been employed for the isolation of selective bioactive compounds [50,58], with each extract exhibiting specific activity. As polar molecules, the aqueous extract contains functional concentrations of nucleosides and polysaccharides. In contrast, alcoholic extracts are rich in nucleosides, polysaccharides, and proteins with a high antioxidant potential. Some of the bioactive components from *Cordyceps* species have been summarized in Table 1.

The neuroprotective mechanisms of the different types of *Cordyceps* extracts have been discussed below.

### 3.1. Cordyceps militaris

*C. militaris* is a valuable TCM that grows on moth larvae (*Lepidoptera*). This fungus has been reported to treat respiratory, renal, hepatic, and cardiovascular diseases and has antiaging, antiviral, anti-inflammatory, and antitumor potentials [59]. Recently, *C. militaris* has become an economical alternative to *C. sinensis* in TCM because it can be easily cultivated under artificial conditions using diverse media [57]. Analyses of the compositions revealed that the concentrations of cordycepin and polysaccharides in the media of cultured *C. militaris* were higher than those in *C. sinensis* from the natural site [60]. The major bioactive components of *C. militaris* are nucleosides (adenosine, uridine, and cordycepin), myriocin, ergosterol, polysaccharides, L-arginine, and L-proline [43,57,61]. Previous studies have shown the presence of GABA (γ-aminobutyric acid), ergothioneine, D-mannitol (cordysepic acid), glycolipids, glycoproteins, xanthophylls (like carotenoids), sterols, statins, phenolic compounds, vitamins, and biominerals in *C. militaris* [60,62]. A previous study reported differences in the concentrations of cordycepin, cordycepic acid, and ergothioneine between fruiting bodies and mycelial biomass. The concentrations of cordycepin, cordycepic acid, and carbohydrates are higher in mycelial biomass, whereas those of ergothioneine and total amino acids are higher in fruiting bodies [62]. The reported optimal drying temperature for *C. militaris* is 60 °C, over which, cordycepin and phenolic compounds are lost [63]. Pentostatin, used as an antileukemia drug, is also produced by *C. militaris* through the same biosynthetic gene cluster for cordycepin production [64]. Similar to other chemotherapeutic drugs, it also has side effects such as diarrhea, nausea, and neurological toxicities [65]. Cordymin is an antifungal peptide that inhibits the mycelial growth of various fungi, including *Candida albicans*, *Bipolaris maydis*, and *Rhizoctonia solani* [66]. Ergosta-7,9(11),22-trien-3β-ol isolated from *C. militaris* shows anti-inflammatory and antioxidative activity [67].

Selective deterioration of cholinergic neurons in AD diminishes acetylcholine (ACh) levels, contributing to cognitive decline [68]. In addition to acting as a neurotransmitter, ACh also induces neurite outgrowth [69,70]. The methanolic extract of *C. militaris* promoted neurite outgrowth and ACh expressions in Neuro 2A mouse neuroblastoma cells in a dose-dependent manner (5–20 µg/mL). It also reversed scopolamine-induced memory deficits in rats and increased central cholinergic function at a dose of 300 mg/kg [71]. The ethanolic extract has been known to promote neurite outgrowth in Neuro 2A cells [72], provide protection from amyloid beta (Aβ)-induced toxicity [73], reduce the expression of inflammatory markers (cyclooxygenase-2 (COX-2) and inducible nitric oxide synthase (iNOS)), and downregulate mitogen-activated protein kinases/c-Jun *N*-terminal kinase/extracellular signal-regulated kinase (MAPK/JNK/ERK) pathway in C6 glial cells [74], which helped to reduce stress, inflammation, and apoptosis [75]. In addition, the extract restores recognition and memory functions by inhibiting oxidative stress (nitric oxide (NO) and lipid peroxidation) caused by toxic peptides [76]. Moreover, it upregulated the dopaminergic system in vivo and in vitro by upregulating tyrosine hydroxylase, an enzyme that catalyzes the rate-limiting steps in the biosynthesis of dopamine and other catecholamines [77].

One of the most conspicuous age-related diseases is ischemia, which is a common form of neurodegeneration that leads to cognitive impairment in the elderly [78]. The post-ischemic brain induces hippocampal neuronal death, neuroinflammation, and neuropathy, similar to AD [79]. Post-ischemic treatment with the butanolic extract of the fungus (WIB-801C: 50 mg/kg) decreased the inflammatory cell infiltration into ischemic lesions by inhibiting chemotaxis through adenosine receptor A3 (A3AR), thus providing neuroprotection in the middle cerebral artery occlusion (MCAO) rat model [80]. Moreover, after spinal cord injury (SCI), it mitigated blood–spinal cord barrier (BSCB) disruption by inhibiting matrix metalloprotease-9 (MMP-9), downregulating the expression of chemokine and promoting that of pro-nerve growth factor (NGF) in microglia (MG) [81]. The fungus also improved memory impairment caused by global cerebral ischemia and memory deterioration by delaying neuronal death, decreasing MG expression in the CA1 region of the hippocampus in rats [82], and increasing the expression of brain-derived neurotrophic factor (BDNF) and tyrosine kinase B (TrkB) in gerbils [83].

*C. militaris* aqueous extract showed beneficial effects in a D-galactose (Gal)-induced aging mouse model by improving memory [84]. Extract supplementation improved the levels of antioxidants (superoxide dismutase (SOD), glutathione peroxidase (GPx), and glutathione (GSH)) and reduced malondialdehyde (MDA) and monoamine oxidase (MAO), which play important roles in the progression of aging. These results suggest a role for the antioxidant action of the fungus in recovering memory impairments in mice with D-Gal-induced aging [84].

In a recent study, nanoencapsulated *C. militaris* extract relieved neuronal pathology in SH-SY5Y cells (human neuroblastoma cells) by significantly improving dopamine secretion and the expression of dopaminergic-specific genes such as leucine-rich repeat kinase 2 gene (LRRK2), LIM homeobox transcription factor 1 beta (LMX1B), Forkhead Box (FOXA2), engrailed homeobox 1(EN1), and nuclear receptor-related 1 protein (NURR1) [85]. In line with this, *C. militaris* treatment enhanced the expression of neuronal protein paired box 6 (PAX6), a crucial player in brain development and function [86], and neuron-specific class III beta-tubulin (nestin), a marker of neuronal progenitor cells in the adult brain [87], indicating the role of *C. militaris* in enhancing neuronal maturation. Furthermore, it reduced amyloid precursor protein (APP) secretion by promoting autophagy [85]. As autophagy helps clear Aβ and tau aggregates in brain cells [88], *C. militaris* is considered important in AD treatment. The downregulated expression of AD-related genes presenilin 1 (PSEN1), presenilin 2 (PSEN2), and APP and the increased expression of the non-amyloidogenic pathway, ADAM metallopeptidase domain 10 (ADAM10), and sirtuin1 (SIRT1) by nanoencapsulated *C*. *militaris* extract suggest its potential in improving AD pathology at both the gene and protein levels [85].

These results suggest that the fungus is highly effective in protecting against memory-related neuronal degeneration in the brain and in retarding the progression of memory deficits associated with various NDs by its antioxidant, anti-inflammatory, and anti-apoptotic properties.

### 3.2. Cordyceps ophioglossoides

*C. ophioglossoides*, commonly known as the “golden thread *Cordyceps*”, is colonized on fruiting bodies of truffle-like *Elaphomyces* [89]. The fungus contains a variety of polysaccharides (antioxidant nature), ophiocordin (antibiotic), peptibiotics (antibiotic and antifungal properties), sesquiterpenes (antitumor activity), balanol (a protein kinase inhibitor with antitumor activity), and arsenocholine-O-sulfate (a nontoxic form of arsenic) [89,90,91,92,93].

Aβ_(25–35)_ represents the biologically active region of Aβ, since it is the shortest fragment that displays large β-sheet aggregated structures, keeping the toxicity of the full-length peptide [94]; hence, it is often used as a model for inducing toxicity and memory deficits. The neuroprotective effect of *C. ophioglossoides* (methanolic extract) has been observed in vitro (extract: 100 μg/mL) and in vivo (extract:100 mg/kg) in Aβ_(25–35)_ AD models, where the fungal extract protected SK-N-SH human neuroblastoma cells from cell death and helped in the restoration of spatial memory loss in induced memory deficit by Aβ_(25–35)_ in rats probably by suppressing Aβ-induced oxidative stress [95].

### 3.3. Cordyceps sinensis

*C. sinensis* is the most popular *Cordyceps*, which parasitizes the larva of *Hepialus armoricanus*. This fungus has long been used in TCM to promote longevity and has anti-inflammatory and antitumor activities [24]. The major biochemical markers of nucleosides are adenosine and cordycepin [50,96], with immunomodulatory and antioxidant activities. In 2008, Yuan et al. reported the presence of other nucleosides (thymine, adenine, cytosine, uracil, uridine, hypoxanthine, ionosine, guanosine, and thymidine) in aqueous extracts of *C. sinensis* [97]. Polysaccharides are major contributors to the biological activities of *C. sinensis*. Guan et al. identified several monosaccharides (fructose, mannitol, galactose, arabinose, ribose, rhamnose, mannose, xylose, glucose, and sorbose) using GC-MS [98]. Ergosterol is the main identified sterol [99] and is present either as free or esterified ergosterol [50,100,101] with antitumor activity [102]. Other compounds, such as polyamines and free fatty acids, have also been identified in *C. sinensis* extracts [103]. Two peptides (cordymin and cordycedipeptide) and an ergosterol (H1-A) with biological activities were also isolated from the fungus [104].

Aqueous and different alcoholic extracts (CSEs) from the fungus revealed the presence of the antioxidants hesperidin, rutin, and ascorbic acid by high-performance thin-layer chromatography (HPTLC). Hesperidin, rutin, and ascorbic acid were present at high concentrations in the aqueous extract. However, the highest hesperidin content was observed in the 25% alcoholic extract in comparison to others [105]. Additionally, adenosine, adenine, and uracil are present at higher concentrations in the aqueous extract than in the other extracts [106]. The protective effects of the extracts against hypoxia-induced oxidative stress and inflammation were studied in mouse hippocampal (HT22) cells. CSEs (250 μg/mL) show neuroprotection by increasing the expression of endogenous antioxidants (GSH, GPx, and SOD), limiting lipid oxidation by decreasing MDA levels and reducing the level of inflammatory cytokines interleukin-6 (IL-6) and tumor necrosis factor-α (TNF-α) as well as transcription factor nuclear factor-κB (NF-κB) to various extents. The aqueous extract is more effective as an antioxidant in hypoxia, whereas the alcoholic extract prevented oxidative stress and inflammation [105], owing to the presence of more phenolics and flavonoids [106].

The aqueous [107] and ethanolic [108] extracts of the fungus were also evaluated for anti-inflammatory effects in an experimental middle cerebral artery occlusion/reperfusion (MCAO/R) model, as ischemic brain injury is associated with inflammatory reactions. In addition, neuronal apoptosis is triggered by increased expression of Bcl2-associated X (Bax), an apoptosis regulator, which in turn activates caspase-3 and inhibits B-cell lymphoma-2 (Bcl-2) expression [109]. The extract provided neuroprotection by downregulating the expression of inflammatory cytokines and other inflammatory mediators (IL-1β, TNF-α, Myeloperoxidase (MPO), and adhesion molecules ICAM-1, COX-2, and iNOS), blocking polymorphonuclear cell (PMNC) infiltration, thereby subsiding neurological deficits and infarct volume. Moreover, the aqueous extract also revealed the decreased Bax, cytochrome c (Cyt c), and caspase-3 protein expressions, which in turn improved mitochondrial membrane potential (ψ_m_), thus modulating the electron transport chain in the mitochondria in vivo and in vitro [110].

Multiple sclerosis (MS) is an autoimmune demyelinating disease of the CNS that involves a variety of immune cells [111]. Inflammation resulting from MS is mediated by the infiltration of autoreactive T cells into the CNS through the blood–brain barrier (BBB) [112]. Amongst the T cells, primarily interferon-gamma (IFN-γ)-producing T-helper 1 (Th1) cells and IL-17-producing Th17 cells had an important role in the pathogenesis of the disease [113]. Effector molecules secreted by Th1 cells directly affect the phenotype, function, and recruitment of MG, whereas Th17 cells upregulate chemokines during the inflammatory process [114]. *C. sinensis* extract was reported to reduce the number of Th1 cells in a mouse model of MS/experimental autoimmune encephalomyelitis (EAE), thus relieving EAE severity and the associated pathology [115].

The neuroprotective effect of fermented fungus powder (Cs-C-Q80 or ‘corbrin capsule’) was evaluated in subcortical ischemic vascular dementia induced in a mouse model of right unilateral common carotid artery occlusion (rUCCAO) [116], which damaged the white matter region in the brain, resulting in myelin loss, glial activation, neuroinflammation, and dementia [117]. However, both the prophylactic and therapeutic administration of corbrin (1 g/kg) significantly reduced white matter lesions and improved learning and memory loss through anti-inflammatory actions [116]. A lower dose of corbrin (1 mg/kg) was effective in reducing the pro-inflammatory cytokines (TNF-α, IL-1β, IL-6), improving the levels of oxidative stress parameters (SOD, MDA), increasing ATP concentration, and alleviating neurological deficits in an MCAO mice model [118].

### 3.4. Cordyceps cicadae

*C. cicadae* is the oldest known therapeutic fungus that feeds on *Lepidoptera* species larvae [119]. It has been used in TCM for the treatment of asthma, cancer, convulsions, dizziness, palpitations, and chronic renal disease. Natural *C. cicadae* is a slow-growing fungus in high demand, whereas its anamorph, *Paecilomyces cicadae,* can be cultured easily and used as a substitute for *C. cicadae* to accommodate market requirements [120]. Various bioactive compounds, such as cyclopeptides, myriocins, polysaccharides, nucleosides, and mannitol have been identified in *C. cicadae* [120,121]. LC-MS analyses have detected adenosine and adenosine analogs, N6-(2-hydroxyethyl)-adenosine (HEA), a Ca^2+^ antagonist, and an anti-inflammatory agent [122,123,124]. HEA is a major bioactive compound in *C. cicadae* that exhibits antidiabetic, sedative, analgesic, antitumor [125], and renoprotective activities [126,127]. Another isolated bioactive compound, ergosterol peroxide, exhibits immunomodulatory and anti-inflammatory effects [128,129].

**Table 1:** Some important compounds from *Cordyceps* species and their biological activities.

Bioactive Compound	*Cordyceps* Species	Chemical Class	Biological Activity	References
Adenosine	*C. sinensis*	Nucleoside	Prohibits cancer cell growth Anti-inflammatory effect	[103]
Cordycepin	*C. sinensis* *C. militaris* *C. cicadae*	Derivative of the nucleoside adenosine	Enhances immunity Anti-tumor activity Anti-inflammatory Antimicrobial activity	[57,103,121]
D-mannitol	*C. militaris*	Sugar alcohol	Diuretic effects	[57]
GABA	*C. militaris*	Primary amine	Neurotransmitter	[57]
Ergotheoneine	*C. militaris* *C. cicadae*	Thiourea derivative of histidine	Antioxidant	[57,121]
Lovastatin	*C. militaris*	Statin	Cholesterol-lowering agent	[57]
Uridine	*C. militaris*	Nucleoside	Maintenance of the cellular metabolism	[61]
*N*-(2-Hydroxyethyl) adenosine	*C. cicadae*	Derivative of the nucleoside adenosine	Anti-inflammatory activity	[121]
Cordycepic acid	*C. cicadae*	Sugar alcohol	Bacteriostatic activity	[103,121]
	*C. sinensis*		Antioxidant	
Beauvericin	*C. cicadae*	Cyclic hexadepsipeptide	Antimicrobial and antitumor activity	[121]
Methyl-2-(5-(3-Hydroxybutyl)furan-2-yl)acetate	*C. cicadae*	Furane methyl ester	Anti-AChE activity	[121]
α-furoic acid	*C. cicadae*	Carboxylic acid	Anti-AChE activity	[121]
2-(5-(3-Oxobutyl)furan-2-yl) acetate	*C. cicadae*	Furane methyl ester	Anti-AChE activity	[121]
Hercynine	*C. cicadae*	Histadine derivative	Antioxidant	[121]
EPSF	*C. sinensis*	Polysaccharide	Antioxidant, antitumor	[103]
APS	*C. sinensis*	Polysaccharide	Antioxidant	[103]
CPS-1	*C. sinensis*	Polysaccharide	Antioxidant	[103]
CPS-2	*C. sinensis*	Polysaccharide	Inhibits cell proliferation	[103]
Ergosterol	*C. sinensis*	Phytosterol	Antimicrobial activity Cytotoxicity	[103]
Cordymin	*C. sinensis*	Peptide	Antidiabetic	[103]
Tryptophan	*C. sinensis*	Amino acid	Sedative effects	[103]

Trauma to the CNS and NDs initiate a torrent of cellular and molecular reactions that result in neuronal loss and regenerative failure. To understand the associated mechanisms, the rodent optic nerve crush (ONC) model can be used and later extrapolated to NDs [130]. *C. cicadae* mycelium extract provided neuroprotection in the ONC rat model through anti-apoptotic and anti-inflammatory effects by improving retinal ganglion cell (RGC) density and P1-N2 amplitude [131], which intensified with visual–spatial attention in the visual cortex. The butanol fraction protected rat adrenal pheochromocytoma (PC12) cells against glutamate-induced oxidative damage. Additionally, the extract restored the mitochondrial function, suppressed ROS accumulation, upregulated the antioxidant enzymes (GPX and SOD), increased cell viability, decreased lactase dehydrogenase (LDH) release, and reduced apoptosis [132,133]. Subsequently, adenosine was identified as the main nucleoside responsible for this neuroprotective action [133]. The anti-inflammatory activities of three bioactive nucleosides (adenosine, cordycepin, and HEA) isolated from wild-type and artificially cultured *C. cicadae* were evaluated. Cordycepin was found to be more potent than other nucleosides in limiting the release of pro-inflammatory cytokines by lipopolysaccharide (LPS)-stimulated RAW 264.7; however, no synergistic effect of the three compounds was observed. LPS-induced pro-inflammatory responses were attenuated by HEA through the suppression of the toll-like receptor (TLR)-4-mediated NF-κB signaling pathway [134]. The effects of the hydroalcoholic fungal extract on cisplatin toxicity have also been evaluated. Cisplatin is an anticancer agent involved in multi-organ toxicity, including neurotoxicity. It accumulates in the dorsal root ganglion (DRG) and causes oxidative stress, neuronal apoptosis, and inflammation [135]. The nucleoside-rich extract of *C. cicadae* ameliorated memory impairment and neuropathy by reducing oxidative stress, acetylcholinesterase enzyme (AChE) levels, and inflammation in cisplatin-treated rats [136].

In a recent study, increased levels of bioactive compounds were obtained from cultured *C. cicadae* in deep ocean water (DOW) and minerals, thus increasing their therapeutic value [137]. The effect of DOW-cultured fungus (DCC) was investigated on D-Gal-induced brain damage and memory impairment in rats. DCC (100–500 mg/kg), in turn, improved cognition by alleviating the expressions of antioxidants and inflammatory genes (iNOS, TNF-α, IL-6, IL-1β, COX-2), along with reduced expressions of the aging-related proteins (glial fibrillary acidic protein (GFAP) and PS1) [137].

The neuroprotective activity of *Cordyceps* extracts has been summarized in Table 2.

**Table 2:** Neuroprotective mechanism of *Cordyceps* extracts.

Species	Extract	Study	Model	Study Outcome	Mechanism	Refs.
*C. militaris*	EtOH	in vitro, in vivo	Aβ_(1–42)-_ induced toxicity in mice and C6 glial cells	Improved cognition, decreased NO and lipid peroxidation, downregulated COX-2 and iNOS, downregulated MAPK/JNK/ERK pathway	Antioxidant, Anti-inflammatory	[72,73,74,76]
*C. militaris*	MeOH	in vitro, in vivo	Neuro 2A, scopolamine-induced memory loss in rats	Promoted neurite outgrowth, increased ACh, improved memory	Increase ACh, neurogenesis	[71]
*C. militaris*	AQ	in vivo	Cerebral ischemia-induced short-term memory impairment in gerbils	Protected neuronal death Increased BDNF and TrkB expression	Anti-apoptotic Antioxidant	[83]
*C. militaris*	AQ	in vivo	D-Gal-induced aging mice	Increased SOD, GPx, GSH Decreased MDA Restoration of memory	Antioxidant	[84]
*C. militaris*	EtOH	in vitro, in vivo	PC12 cells and rat	Increased tyrosine hydroxylase	Upregulation of the dopaminergic system	[77]
*C. militaris*	BuOH	in vivo	MCAO-rat, scopolamine-induced memory loss in rats, spinal cord injury	Inhibited MMP-9, downregulated chemokines, delayed neuronal death	Anti-inflammatory	[80,81]
*C. militaris*	AQ	in vivo	Ischemia-induced death and cognitive impairment in rats	Decreased microglial expression Memory improvement	Anti-inflammatory	[82]
*C. militaris*	NP	in vitro	SH-SY5Y	Enhanced the expression of neuronal proteins Increased expression of dopaminergic-specific genes Decreased expression of PS1, PS2, APP Upregulated ADAM10 and SIRT1 Decreased Aβ secretion	Autophagy, neurogenesis, secretion of dopamine	[85]
*C. ophioglossoides*	MeOH	in vitro, in vivo	Aβ _(25–35)-_induced SK-N-SH and rats	Decreased oxidative stress Restored memory	Antioxidant	[95]
*C. sinensis*	AQ, EtOH	in vitro	Hypoxia-induced oxidative stress in HT22	Increased SOD, GPx, GSH Decreased MDA, IL-6, TNF-α, NF-kB	Antioxidant Anti-inflammatory	[105]
*C. sinensis*	AQ, EtOH	in vivo	MCAO/R	Decreased IL-1β, TNF-α, MPO, ICAM-1, COX-2 and iNOS Suppressed PMNC infiltration	Anti-inflammatory	[107,108]
*C. sinensis*	AQ	in vitro, in vivo	MCAO/R	Decreased Bax, Cyt c, Caspase-3	Anti-apoptotic	[110]
*C. sinensis*	-	in vivo	Mice mode of MS-EAE	Decreased Th1	Immunoregulatory	[115]
*C. sinensis*	Fermented	in vivo	rUCCAO mice model	Reduced white matter lesion	Anti-inflammatory	[116]
*C. sinensis*	Fermented	in vivo	MCAO	Decreased TNF-α, IL-1β, IL-6 Increased SOD and ATP Decreased MDA Memory improvement	Antioxidant Anti-inflammatory	[118]
*C. cicadae*	-	in vivo	ONC rat model	Improved retinal ganglion cell density and P1-N2 amplitude	Antioxidant Anti-apoptotic	[131]
*C. cicadae*	BuOH	in vitro	Glutamate induced toxicity in PC12 cells	Increased GPx, SOD Increased cell viability, decreased LDH	Antioxidant Anti-apoptotic	[132,133]
*C. cicadae*	AQ, MeOH	in vitro	LPS-stimulated RAW 264.7 macrophages	Suppressed TLR-4-mediated NF-kB pathway	Anti-inflammatory	[134]
*C. cicadae*	HA	in vivo	Cisplatin-induced toxicity in mice	Reduced IL-6, TNF-α, and IL-1β; decreased AChE and oxidative stress	Antioxidant Anti-inflammatory	[136]
*C. cicadae*	DOW-cultured	in vivo	D-Gal-induced brain damage and memory impairment in rats	Decreased expression of GFAP, PS1 Decreased COX-2, TNF- α, IL-6, IL-1β	Antioxidant Anti-inflammatory	[137]

Abbreviations: AQ: aqueous; MeOH: methanol; EtOH: ethanol; BuOH: butanol; HA: hydroalcoholic; NP: nanoparticle; DOW: deep Ocean water.

## 4. Neuroprotective Potential of Bioactive Compounds from *Cordyceps*

**Figure 1:**
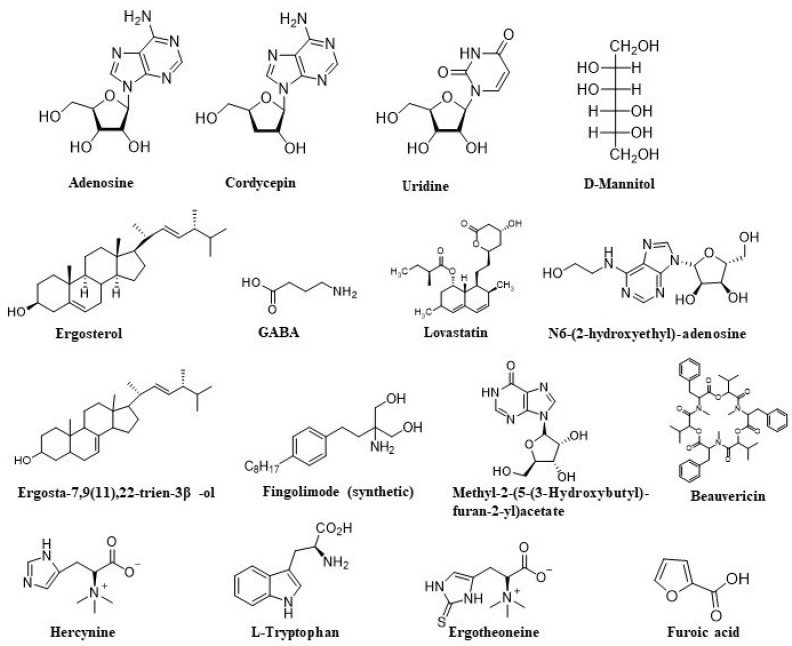
Some of the bioactive compounds from Cordyceps.

*Cordyceps* is a rich source of over 200 bioactive compounds, including nucleotides, nucleosides, polysaccharides, proteins, sterols, vitamins (Vit E, K, B1, B2, and B12), and trace elements [138]. These bioactive compounds are associated with a range of pharmacological activities, including antimicrobial, anti-allergic, antidiabetic, analgesic, anti-apoptotic, anticancer, anti-inflammatory, antioxidant, antiaging, and immunomodulatory effects [23,50,52]. However, few studies have investigated their neuroprotective activities. The mechanisms underlying the neuroprotective effects of these bioactive compounds (Figure 1) under various conditions are discussed below.

### 4.1. Cordycepin

Cordycepin (C_10_H_13_N_5_O_3_, MW 251.24 g/mol) is a bioactive nucleoside (3′-deoxyadenosine) from Cordyceps. It has several pharmacological properties, including anti-aging, anti-inflammatory, anticancer, and antioxidant properties [139,140,141,142,143]. Cordycepin is the most extensively studied bioactive compound in Cordyceps and its neuroprotective activities in different diseases are discussed below.

#### 4.1.1. Neuroprotection in PD

PD is a progressive neurological disorder primarily affecting dopaminergic neurons in the substantia nigra region and is characterized by intracellular α-synuclein aggregates in the form of Lewy bodies and Lewy neurites. The biochemical processes implicated in PD include neuroinflammation, mitochondrial dysfunction, and faulty protein clearance [144].

The neuroprotective effects of cordycepin in PD have been investigated in several neurotoxin-induced models, namely, 6-hydroxydopamine (6-OHDA)-, 1-methyl-4-phenyl-1,2,3,6-tetrahydropyridine (MPTP), and rotenone-induced models, to reiterate disease pathology both in vitro and in vivo. Isolated cordycepin from C. cicadae protected the PC12 cells against 6-OHDA-induced neurotoxicity by reducing caspase-3 activity, improving ψ_m,_ and elevating the levels of antioxidant enzymes [145], and finally, through an anti-apoptotic mechanism via Bax downregulation [146]. Furthermore, cordycepin protected dopaminergic neurons from death and inflammation by inhibiting dynamin-related protein-1 (Drp-1)-mediated (NOD)-like receptor protein 3 (NLRP3) inflammasome activation by increasing AMP-activated protein kinase (AMPK) phosphorylation in a rotenone-induced PD rat model and cultured PC12 cells [147]. Cordycepin alleviated MPTP-induced PD through TLR/NF-κB inhibition and mitigated the cytotoxic effects of MG on LPS-induced PC12 cells [148]. The anti-inflammatory role of cordycepin, along with the neuroprotection, was reported in LPS-induced MG activation in hippocampal cultured neurons (BV2) by assisting neural growth and development in the hippocampal neurons and downregulating the levels of TNF-α, IL-1β, iNOS, and COX-2, leading to an anti-inflammatory effect [149]. As TNF-α and IL-1β had a role in the activation of NF-κB, COX-2 was in turn activated by it [150], suggesting the involvement of the NF-κB pathway in the anti-inflammatory action of cordycepin. Therefore, constraining the TLR-4/NF-κB pathway and NLRP3 inflammasome activation would be valuable therapeutic targets for controlling pyroptosis and consequently advancing neurodegeneration in PD [151].

Glutamate is a key excitatory neurotransmitter that maintains cognitive, motor, and sensory functions, while GABA is an inhibitory neurotransmitter that maintains neuronal function. An imbalance between the glutamate and GABA synaptic systems results in impaired neural function that affects memory and cognition [152]. The extra synaptic diffusion of glutamate is strongly associated with MG activation and neuroinflammation, which are considered common characteristics in many NDs, including PD [153]. Moreover, hyperactivations of postsynaptic glutamate receptors α-amino-3-hydroxy-5-methyl-4-isoxazole-propionic acid (AMPA) and *N*-methyl-d-aspartic acid (NMDA) were implicated in various neurological dysfunctions. Hence, the antagonists of these receptors have several beneficial effects in reversing motor symptoms [154]. Cordycepin suppressed AMPA and NMDA-receptor-mediated responses through a reduction in the presynaptic mechanism [155] and reduced the frequency of glutamatergic and GABA-ergic postsynaptic transmission without affecting the amplitude through A1AR activation [156]. This mechanism might provide neuroprotection against Aβ toxicity, hypoxia, ischemia, and other excitotoxic disorders. Cordycepin also protects against glutamate-induced oxidative toxicity in HT22 cells by downregulating the endoplasmic reticulum (ER) stress-specific caspase-12, which is important for the initiation of ER stress-induced apoptosis. Additionally, cordycepin inhibited the expression of pro-apoptotic genes (C/EBP homologous protein (CHOP) and Bax) and genes involved in ER stress-induced apoptosis (JNK, protein kinase R (PKR)-like ER kinase (PERK), and mitogen-activated protein kinases (p38)) [157]. Cordycepin also reduced ROS and Ca^2+^ levels. A1AR activation has been shown to mediate the neuroprotective effect of cordycepin [157,158].

Promising results from various studies have indicated the potential of cordycepin as a drug for the treatment of PD.

#### 4.1.2. Neuroprotection in AD

AD is a multifactorial ND characterized by neuronal loss, accumulation of Aβ plaques, and neurofibrillary tangles. Aβ toxicity is accompanied by increased ROS production in neurons, which in turn leads to a series of events, like AChE activation, rise in Ca^2+^ concentration, mitochondrial dysfunction, and increased neuronal apoptosis resulting in cognitive deficit [159,160]. AChE hydrolyzes ACh, a neurotransmitter required for synaptic transmission, and, in turn, increases AChE activity, adversely affecting neurotransmission. The neuroprotective effect of cordycepin has been established in Aβ-induced rat hippocampal cells in the AD model [158]. Cordycepin effectively reduced ROS production and Ca^2+^ levels, inhibited AChE, suppressed apoptosis, and downregulated p-tau expression. These results suggest the involvement of A1AR in the neuroprotective activity of cordycepin.

Communication between neurons and MG in healthy brains and NDs has been studied extensively [161]. Two forms of active MG with antagonistic actions have been identified: MG-M1 (pro-inflammatory) and MG-M2 (anti-inflammatory); the MG-M1 form is prevalent in AD, leading to Aβ and tau accumulations, neuronal damage, and synaptic dysfunction [162]. Since MG polarization determines the fate of MG neurons in NDs, it is an efficient approach to fight AD. Recently, cordycepin was reported to induce MG-M2 polarization through the activation of cAMP-response element binding protein (CREB) activation and upregulation of NGF. This mechanism improved cognitive deficits in an APP/PS1 mouse model [163].

#### 4.1.3. Neuroprotection in Ischemic Stroke

Cerebral ischemia/reperfusion injury (CI/RI) is a general feature of ischemic stroke, involving an interval of limited blood flow to the brain, followed by the refurbishment of blood supply through clinical intervention [164]. CI/RI results in neuronal injury or death, and irreversible brain damage due to oxidative stress, amino acid toxicity, Ca^2+^ overload, BBB dysfunction, inflammation, and apoptosis [165]. Cordycepin has been reported to exert protective effects in vitro and in vivo against CI/RI by restoring the levels of oxidative stress markers (MDA and SOD), reducing the levels of excitatory amino acids (glutamate and aspartate), and suppressing the expression of the inflammatory enzyme MMP-3 [139]. A study showed that a lower dose (10 mg/kg) of cordycepin was more effective in multiple myeloma cells, indicating that a higher concentration might induce cell death by preventing RNA synthesis [166] in astrocytes, reducing the number of astrocytic glutamate transporters and elevating extracellular glutamate levels. In addition, both C. militaris (water extract, 500 mg/kg) and cordycepin (10 mg/kg) reduced the levels of 4-hydroxynonenal (a lipid peroxidation marker) by reducing oxidative stress [167], delaying membrane depolarization, adjusting the electrophysiological activity of hippocampal CA1 neurons [168,169], and improving learning and memory [170] through A1AR activation [171].

#### 4.1.4. Neuroprotection in Multiple Sclerosis

MS is a prominent neuroinflammatory autoimmune disorder characterized by the intrusion of immune cells from the perivascular region into the CNS. It is a demyelinating disease in which myelin is lost from various regions, leaving a scar (sclerosis) and disrupting signal transmission to and from the brain [172]. Cordycepin promoted remyelination by suppressing neuroinflammation (IL-6 and IL-1β) and upregulation of BDNF and anti-inflammatory cytokines (IL-4, IL-10, and TGF-β) in the corpus callosum and hippocampus in a cuprizone (CPZ)-induced mice model [173]. Cordycepin suppressed LPS-induced dendritic cell activations by mitigating oxidative stress through alleviating the protein kinase B/extracellular signal-regulated kinase/NFκB (AKT/ERK/NF-kB) signaling pathways in vitro and decreased the levels of migration/adhesion molecules (integrin β1, integrin α4, c-type lectin, intermolecular adhesion molecule-1 (ICAM-1), CC motif chemokine receptor 7 (CCR7)) in vitro and in vivo. Furthermore, cordycepin treatment in the experimental autoimmune encephalomyelitis (EAE) mice model decreased the level of chemokines (CC chemokine ligand 6 (CCL6), PAR response elements 2 (PARRES-2), IL-16, C-X-C motif chemokine ligand 10 (CXCL10), and cc motif chemokine ligand 12 (CCL12)) in the CNS and spinal cord and inhibited the production of pro-inflammatory cytokines (IFN-γ, IL-6, TNF-α, and IL-17) in activated microglial cells, macrophages, and Th cells in vitro [174], thus potentially ameliorating MS progression.

In summary, cordycepin ameliorates motor dysfunction, improves remyelination, decreases the number of glial cells, suppresses pro-inflammatory cytokines, increases anti-inflammatory cytokines and neurotrophic factors, and reduces oxidative stress. Consequently, it is a potential candidate for treating demyelination-associated diseases such as MS.

#### 4.1.5. Neuroprotection in Traumatic Brain Injury

Traumatic brain injury (TBI) leads to serious neurological dysfunctions that affect motor and cognitive functions. Additionally, white matter injury (WMI) resulting from secondary TBI is extremely vulnerable to neuroinflammation owing to a vicious cycle caused by the penetration of neuroinflammatory receptor immune cells. TLR-4 is expressed on astrocytes, microglia, and neurons and is activated upon infection, brain injury, BBB disruption, and many NDs, including AD, PD, and ALS [175,176]. Another important protein is MMP-9, which contributes to BBB disruption and edema after TBI [177]. Hence, therapies directed toward these targets are beneficial.

The administration of cordycepin in TBI mice (10 mg/kg) [178] and rats (20 mg/kg) [179] ameliorated long-term neurological deficits, as observed in behavioral tests. Immunohistochemical staining indicated that cordycepin secured the number and structure of nonmyelinated and myelinated axons, thereby enhancing their conductive abilities. Cordycepin administration also decreased the levels of pro-inflammatory markers: cluster of differentiation-16 (CD16), interleukin-17α (IL-17α), IL-1β, iNOS, and MPO, with an upregulation of anti-inflammatory ones (CD-206, IL-10, Arginase-1). Moreover, it elevated the expression of tight-junction proteins (zonula occludens-1 (ZO-1) and occludin) and reduced the activity of MMP-2 and MMP-9, preserving the integrity of the BBB [178,179]. Additionally, cordycepin inhibits NADPH oxidase (NOX1), which is the main contributor to ROS, by disrupting the BBB after TBI [179].

As the brain is sensitive to oxygen levels, hypobaric hypoxia (HH) could lead to neuronal death and neuropsychological dysfunction [180]. HH induces oxidative stress and neuroinflammation, which eventually disturb the integrity of the BBB. Early HH followed by TBI increases the severity of secondary brain injury [181]. Cordycepin (10 mg/kg) inhibits the hippocampus-dependent memory impairment caused by acute HH, relieves hyperactivation of astrocytes/microglia in the CA1 region, and mitigates HH-induced activation of the TLR-4/NF-κB neuroinflammation in the rat model. In addition, it conserved BBB integrity by repressing MMP-9 expression and increasing the levels of tight-junction proteins (claudin-5, occludin, and ZO-1) in the hippocampus [182].

From the above studies, it is evident that cordycepin provides neuroprotection through antioxidant and anti-inflammatory mechanisms via A1AR activation. Additionally, it enables the presynaptic suppression of excitatory synaptic transmission by limiting the release of excitatory neurotransmitters, a novel system for modulating CNS activity.

The mechanism of neuroprotection displayed by cordycepin in various conditions has been summarized in Table 3.

**Table 3:** Neuroprotective mechanism of cordycepin.

Disease	Study Model	Mechanism	MOA	Refs.
PD	6-OHDA-induced neurotoxicity in PC12 cells	Decreased caspase-3, increased SOD and ψ_m_	Antioxidant activity	[145]
	Rotenone-induced toxicity in rat model	Decreased Bcl2 expression, increased ψ_m_, decreased caspase-3	Anti-apoptotic Antioxidant	[146,147]
	MPTP-induced PD in rats and PC12 cells	Suppressed TLR4/NF-κB pathway	Anti-inflammatory	[148]
	Glutamate-induced oxidative toxicity in HT22 cells	Downregulated caspase-12 Deceased expression of CHOP, Bax, JNK, PER, p38 Reduced ROS and Ca^2+^	Anti-apoptotic, Antioxidant, A1AR activation	[157]
	LPS-induced BV2 cells	Neurogenesis Downregulated TNF-α, IL-1β, iNOS, Cox2	Anti-inflammatory Neurogenesis	[149]
	LPS-treated C57BL/6J mice and BV2 cells	Suppressed TLR4/NF-κB-mediated NLRP3 inflammasome activation and GSDMD-related pyroptosis Inhibited pore formation in the plasma membrane Reduced the release of pro-inflammatory mediators	Anti-apoptotic Anti-inflammatory	[151]
	Hippocampal brain slice from rats	Reduced excitatory synaptic transmission	Synaptic transmission	[155]
AD	Aβ-induced toxicity in primary hippocampal neurons	Downregulated pTau, anti-AChE, reduced ROS and Ca^2+^	Anti-apoptotic Antioxidant Enzyme inhibition A1AR activation	[158]
	APP/PS1 mice model	Microglia/macrophage polarization through CREB	Neurogenesis	[163]
Ischemic Stroke	OGD model	Increased SOD Decreased MDA Suppressed Glu and Asp Decreased MMP3	Antioxidant	[139]
	Ischemic damage in gerbils; β-amyloid and ibotenic acid-induced hippocampal CA1 pyramidal neuronal hyperactivity	Reduced 4-hydroxynonenal, delayed membrane depolarization	Antioxidative A1AR activation	[167,168,169,170]
	Acute hypobaric hypoxia-induced BBB disruption and cognitive impairment in rats	Increased tight-junction proteins (claudin5, occluding, zonula occludens-1) Inhibited TLR-4/NF-κB/MMP-9 pathway	Anti-inflammatory Antioxidant	[182]
MS	LPS-induced dendritic cells, MS-EAE mice model	Inhibited AKT/ERK/NF-kB pathway Decreased integrin (β1,α-4), c-type lectin, ICAM1, CCR7 Decreased chemokines Decreased INF-γ, IL-6, IL-17, TNF-α	Antioxidant, Anti-inflammatory	[174]
	CPZ-induced demyelination in mice	Decreased IL-6, IL-1β Increased IL-4, IL-10, and TGF-β Upregulated BDNF Promoted remyelination	Anti-inflammatory	[173]
TBI	TBI-mice, rats	Decreased MMP-2, MMP-9; CD-16, IL-17, NOX1, MPO, iNOS Increased ZO-1, CD-206, IL-10, IL-1β, Arginase-1 Suppressed neutrophil infiltration	Anti-inflammatory Antioxidant	[178,179]

The neuroprotective mechanisms of other bioactive compounds from Cordyceps have been discussed below.

### 4.2. N^6^-(2-Hydroxyethyl)-Adenosine (HEA)

N^6^-(2-hydroxyethyl)-adenosine (HEA), a bioactive nucleoside (C_12_H_17_N_5_O_5_; MW 311.29 g/mol), was identified from the butanolic fraction of C. cicadae. It is a calcium antagonist and interacts with human serum albumin [125,127]. HEA is a potent antioxidant with glucose-lowering, hepatoprotective, cardioprotective, sedative, antitumor, eye-protective, and anti-inflammatory properties [126,134,165,183,184].

Limited literature is available on the neuroprotective activities of HEA. HEA (5–40 μM) protected against H_2_O_2_-induced oxidative stress in PC12 cells by increasing cell viability, decreasing LDH release, preventing ψ_m_ breakdown, limiting ROS generation, inhibiting lipid peroxidation, and reducing inflammatory cytokines (IL-6, IL-1β, TNF-α, and NF-κB) [185]. HEA alleviated the pro-inflammatory response in RAW264.7 macrophages by suppressing the TLR-4/NF-κB pathway in LPS-induced inflammation [134].

### 4.3. Adenosine

Adenosine is a pentose sugar bonded to adenine, that is, adenine riboside (C_10_H_13_N_5_O_4_; MW 267.24 g/mol). It is a neuromodulator of the CNS that primarily operates via the adenosine A1 receptor (A1R). A1R activation plays a neuroprotective role by modulating the Gα/cAMP/PCK pathway, enhancing synaptic plasticity, memory, and cognition [186].

Excessive glutamate triggers intracellular Ca^2+^ influx by activating NMDA receptors and enhancing mitochondrial oxidative stress, which in turn activates mitochondria-associated apoptotic proteins. Furthermore, the upregulation of MAPK during oxidative stress is associated with apoptosis in several NDs. However, adenosine isolated from C. cicadae protected PC12 cells from glutamate-induced apoptosis by increasing the Bcl-2/Bax ratio, decreasing oxidative stress (increasing GPX, SOD, ψ_m_), and alleviating Ca^2+^ overload (via decreasing p38/JNK/ERK phosphorylation) [133]. Hence, adenosine is a promising pharmacophore for the treatment of NDs.

### 4.4. Polysaccharides

Polysaccharides obtained from Cordyceps are also important bioactive compounds with a wide range of activities, including immunomodulatory, antioxidant, and antitumor activities [187,188,189,190,191].

Polysaccharides from the fruiting bodies of C. militaris were evaluated for their protective effects in a D-Gal-induced aging mouse model [192], where they protected mitochondrial integrity by scavenging free radicals and increasing the activity of the antioxidant enzymes responsible for aging. Heterogeneous polysaccharides (CPA-1 and CPA-2), mainly composed of mannose, glucose, and galactose, isolated from C. cicadae provided neuroprotection against glutamate-induced toxicity in PC12 cells [193] by increasing cell viability and the levels of antioxidant enzymes (GSH-Px and SOD) and reducing LDH release, ROS, and Ca^2+^ levels through their antioxidant action. A similar neuroprotective effect was observed in H_2_O_2_-treated PC12 cells by acid polysaccharides (APS) from C. sinensis [194,195]. Furthermore, crude polysaccharides (CPs) and non-digestible polysaccharides (NPs) isolated from cultivated *C. cicadae* exhibited anti-inflammatory activities in LPS-induced RAW264.7 macrophages [196]. Compared to CPs, NPs displayed better inhibition of NO, TNF-α, and IL-1β production in LPS-stimulated cells. The different conformations and molecular weights of the two polysaccharides may be responsible for the variations in their activities. Additionally, CP70, a polysaccharide from *C. cicadae,* displayed anti-aging activity and extended the lifespan of Drosophila by upregulating the expression of antioxidant enzymes (catalase (CAT) and SOD) [197].

In summary, the polysaccharides from Cordyceps provide neuroprotection through antioxidant and anti-inflammatory mechanisms.

### 4.5. Ergosta-7, 9 (11), 22-Trien-3β-ol (EK100)

EK100 (C_28_H_44_O; MW 396.64 g/mol) is a derivative of fungal ergosterol. Ergosterols are the active components of Cordyceps and have important therapeutic activities, such as analgesic, antimicrobial, antitumor, antioxidant, anti-inflammatory, antidiabetic, antihyperlipidemic, and immunomodulatory activities [129,198,199,200,201,202,203].

In the AD model of Drosophila with the pan-neuronal overexpression of human Aβ, EK100 improved the life span, motor functions, and memory by modulating MG activation, only without having any effect on the oxidative stress markers [204]. MG-mediated innate immunity is a double-edged sword, particularly in AD. Activated MG clears Aβ but inevitably damages neurons in the microenvironment [205]. A decline in innate immunity (MG activation) reduces its impact on Aβ clearance, hence leading to Aβ deposition and generating oxidative stress.

During chronic inflammation, the MAPK/activator protein (AP-1) pathway plays a critical role in the release of pro-inflammatory cytokines. Hence, controlling LPS-induced TLR-4/NF-kB/MAPK may benefit cells. The anti-inflammatory potential of ergosterol EK100 isolated from *C. militaris* [206] was studied in LPS-induced RAW264.7 cells. EK100 (80 μM) significantly reduced the cytokine releases and the levels of pro-inflammatory mediator proteins, attenuated phosphatidylinositol-3-kinase (PI3K)/Akt phosphorylation, inhibited the TLR-4/myeloid differentiation factor 88 (MyD88)/IκB kinase (IKK) inflammatory signaling pathway, and suppressed the nuclear translocation of p65 and p50 in the treated cells. Molecular docking studies revealed that EK100 restricted docking of LPS to the LPS-binding protein (LBP), CD14, and TLR-4/myeloid differentiation-2 (MD-2) co-receptors and finally suppressed the TLR-4/NF-kB inflammatory pathway. Moreover, EK100 not only modulated the LPS/TLR-4-related MAPK/AP-1-induced inflammatory pathway but also activated nuclear factor erythroid 2-related factor (Nrf2)/heme oxygenase-1(HO-1) antioxidative signaling by increasing the levels of antioxidant enzymes (GPX, SOD, and CAT) [67].

At present, recombinant tissue plasminogen activator (rtPA) is the only standard treatment for ischemic stroke, which damages the brain tissue through brain infarction and inflammation, [207]. Recently in a study, EK100 (30–120 mg/kg) supplementation ameliorated ischemic stroke brain injury in mice [208]. The combination of EK100 (60 mg/kg) and r-tPA (10 mg/kg) enhanced the protective action compared to either of them alone. Reduced levels of inflammation and apoptosis markers (p65NF-κB and caspase-3) and upregulation of neurogenesis protein (doublecortin) by EK100 via PI3K/AKT activation, glycogen synthase kinase-3 (GSK-3) inhibition, and β-catenin upregulation were observed [208]. GSK-3 inhibition has already been reported as an effective neuroprotective approach to ischemic stroke [209].

Intracerebral hemorrhage (ICH) is a neurological disorder characterized by intensified excitotoxicity, neuroinflammation, and apoptosis in the damaged brain tissue. The upregulation of COX-2 induces an inflammatory cascade by activating specific prostaglandin receptors post-ICH [210]. Furthermore, oxidative stress and MMP-9 activation have deleterious effects on brain injury as they promote DNA damage, apoptosis, and edema [211]. Hence, treatments directed towards controlling the expression of COX-2 and MMP-9 may be beneficial for ICH. EK100 was reported to inhibit JNK/MAPK activation and COX-2 and MMP-9 expression in vitro (BV2 cells) and in vivo (collagenase-induced ICH mice) and improved brain edema and neurobehavioral defects in ICH mice [212].

Therefore, EK100 is a promising novel dual strategy for the treatment of inflammatory diseases that not only suppresses inflammatory transcription factor signaling but also activates the antioxidative transcription factor signaling pathway.

### 4.6. Cordymin

Cordymin is a 10.9 kDa weight fungal peptide with an *N*-terminal sequence of AMAPPYGYRTPDAAQ, isolated from Cordyceps [66,213]. It is reported to possess anticancer, antidiabetic, anti-inflammatory, and antinociceptive activities [214,215].

Ischemia-reperfusion (IR) injury is a common characteristic of ischemic stroke resulting from restoration of blood supply after ischemia. It results in the release of inflammatory cytokines and free radicals, resulting in apoptosis [216]. In the cerebral ischemia-reperfusion injury rat model, cordymin (1–4 mg/kg) protected the ischemic brain by elevating antioxidant activity (through increasing GSH, reducing lipid peroxidation), decreasing inflammation (downregulating IL-1β and TNF-α and C3 protein), and repressing infiltration of polymorphonuclear cells (PMNCs) in the lesion [213]. Consequently, cordymin can be used as a promising protective agent against IR injury.

### 4.7. Active Polypeptide

Active polypeptides are specific protein fragments that have a positive effect on health. Bioactive polypeptides can be directly absorbed into the system and serve as carriers. *C. militaris* polypeptides improve immunity and exhibit antioxidant properties [217,218].

The effect of Cordyceps polypeptide was studied in a scopolamine-induced memory-impaired mouse model, where it improved the condition through antioxidant action (increased SOD and decreased MDA) and retarded AChE activity in the mouse brain. It also elevated the activity of the sodium–potassium pump (Na^+^-K^+^-ATPase), which is involved in the energy supply, and upregulated the expression of GABA and glutamate, which are the central inhibitory and stimulatory neurotransmitters, respectively. Moreover, the polypeptide increased the gene expression of Slc18a2 (secretion of neurotransmitters) with a concomitant decrease in the expression of Pik3r5 (cell proliferation and apoptosis) and Il-1β (pro-inflammatory) [219].

### 4.8. Fingolimod

Fingolimod (FTY-720; C_19_H_33_NO_2_; MW 307.47 g/mol) is a synthetic analog of myriocin (C_21_H_39_NO_6_; MW 401.54 g/mol), which is a non-proteinogenic fungal amino acid.

Myriocin ((2S, 3R, 4R)-(E)-2-amino-3,4-dihydroxy-2-(hydroxymethyl)-14-oxoeicos-6-enoic acid) was isolated from Isaria sinclairii (imperfect stage of C. sinclairii) almost 30 years ago [220]; it shows strong in vitro immunosuppressive activity; however, it induces toxicity in vivo. Extensive chemical modification of the myriocin structure generated fingolimod [FTY-720; (2- amino-2-[2-(4-octylphenyl)ethyl]propane-1,3-diol)], which has stronger immunosuppressive activity and less toxicity than myriocin [221]. The structure of fingolimod is closely related to that of sphingosine; hence, fingolimod is phosphorylated by sphingosine kinases. Phosphorylated fingolimod depletes lymphocyte circulation by activating the sphingosine-1-phosphate receptor (S1PR) and displaying potent immunosuppressive activity [222,223,224]. As S1PR is located in the CNS, the neuroprotective properties of FTY720 have been reported in experimental models of AD and PD [225,226] and granted with US Food and Drug Administration (FDA) approval as the first oral drug to reduce MS relapse [227,228]. Recently, FTY720 was found to exert neuroprotection in CI/RI by reducing the protein levels of IL-17α in the glial cells and reducing inflammatory reactions in the brain. Moreover, it protected the entire neurovascular unit by reducing the infarct volume, protecting the BBB, improving neurological deficits, and reducing apoptosis in the neurons [229].

The mechanism of neuroprotection exerted by bioactive compounds from Cordyceps has been summarized in Table 4.

**Table 4:** Neuroprotective potential of other bioactive components from Cordyceps.

Name	Nature	Study	Model	Study Outcome	Mechanism	Refs.
N^6^-(2-hydroxyethyl)-adenosine	Nucleoside	in vitro	H_2_O_2_-induced oxidative stress in PC12 cells	Reduced IL-6, IL-1β, TNF-α and NF-kB Reduced LDH release, increased Ψ_m_	Antioxidant Anti-inflammatory	[185]
		in vitro	LPS-induced inflammation in RAW264.7 macrophages	Decreased pro-inflammatory cytokines by suppressing TLR-4/NF-kB pathway	Anti-inflammatory	[126]
Adenosine	Nucleoside	in vitro	Glutamate-induced toxicity in PC12 cells	Increased GSH-Px and SOD Increased Bcl-2/Bax ratio Reduced the expression of ERK, p38, and JNK, increased Ψ_m_	Antioxidant Anti-inflammatory Anti-apoptotic	[133]
Mixture	Polysaccharide	in vivo	D-Gal-induced aging mice model	Decreased ROS Increased antioxidant enzymes Protected mitochondria	Antioxidant Anti-aging	[84]
CPA-1, CPA-2	Polysaccharide	in vitro	Glutamate-induced toxicity in PC12 cells	Increased cell viability; ncreased GSH-Px, and SOD Reduced LDH release, ROS, and Ca^2+^ levels	Antioxidant	[193]
CP, NP	Polysaccharide	in vitro	LPS-induced inflammation in RAW264.7 macrophages	Inhibited NO, IL-1β, TNF-α	Anti-inflammatory	[196]
CP70	Polysaccharide	in vivo	*Drosophila*	Increased CAT, SOD expression	Antioxidant Anti-aging	[197]
APS	Polysaccharide	in vitro	H_2_O_2_-induced stress in PC12	Increased cell viability; increased GSH-Px, and SOD Reduced LDH release, ROS, and Ca^2+^ levels	Antioxidant	[194]
Ergosta-7, 9 (11), 22-trien-3β-ol	Ergosterol	in vivo	*Drosophila* AD model	Reduced microglia activation and inflammatory markers	Anti-inflammatory	[204]
		in vitro	LPS-induced RAW264.7 and BV2 cells	Reduced the cytokine release and pro-inflammatory markers Suppressed TLR4/NF-kB pathway, activated Nrf2/HO-1 pathway	Antioxidant Anti-inflammatory	[67,206]
		in vivo	Ischemic stroke brain injury in mice	Increased neurogenesis, upregulated PI3K/AKT pathway	Anti-inflammatory Anti-apoptotic	[208]
		in vivo, in vitro	Collagenase-induced ICH in mice, BV2 cells	Downregulated MMP-9, COX-2	Anti-inflammatory	[212]
Cordymin	Peptide	in vivo	Ischemic stroke brain injury in mice	Elevated GSH Reduced MDA, IL-1β, TNF-α Reduced infiltration of PMNCs	Antioxidant Anti-inflammatory	[213]
Active polypeptide	Peptide	in vivo	Scopolamine-induced memory impairment in mice	Increased SOD, Na-K-ATPase Decreased MDA and AChE Increased secretion of neurotransmitters	Antioxidant Anti-inflammatory Anti-apoptotic	[219]
Fingolimod	Myriocin synthetic analog	in vivo, in vitro	Focal CI/RI in the rat, mice PD model 6-OHDG Rotenone-induced SH-SY5Y Cells	Protected BBB Improved neurological deficits Reduced IL-17 Reduced caspase-3 expression	Immunosuppressant Anti-inflammatory Anti-apoptotic	[225,226,229]

**Figure 2:**
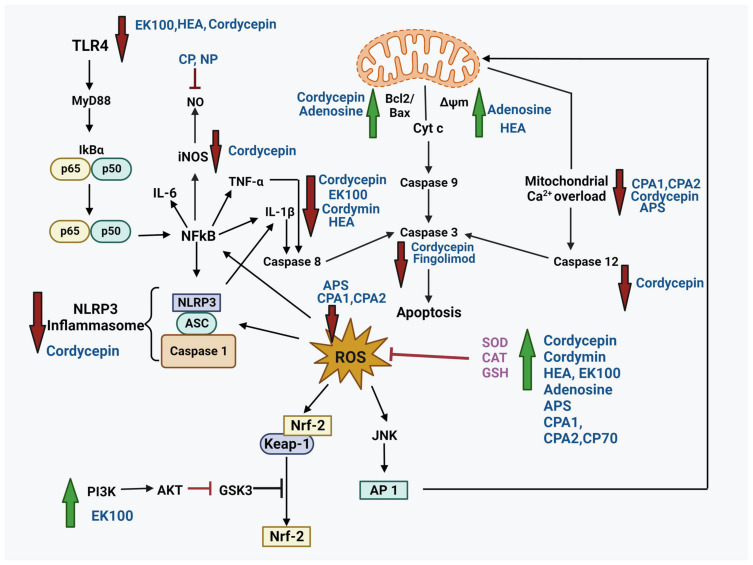
Summary of neuroprotective mechanisms exerted by various bioactive compounds from Cordyceps. Cordycepin, HEA, and EK100 suppressed the TLR-4/NF-kB pathway, suppressing inflammatory cytokines (TNF-α, IL-1β, IL-6). Additionally, cordycepin suppressed NLRP3 inflammasome activation and iNOS levels. Cordymin also inhibited pro-inflammatory cytokines in addition to elevating the levels of antioxidant enzymes. CP and NP inhibited NO levels. Mitochondrial membrane potential was improved by adenosine and HEA. Cordycepin and adenosine improved Bcl2/Bax ratio. CPA1, CPA2, APS, and cordycepin reduced Ca^2+^ overload with cordycepin further decreasing caspase-12 and caspase-3 activity. Fingolimod also reduced caspase-3 indicating an anti-apoptotic mechanism. CPA1, CPA2, and APS decreased ROS. EK100 activated the PI3K/AKT pathway. Cordycepin, HEA, adenosine, APS, CPA1, CPA2, and CP70 increased the antioxidant levels. (red arrows represent downregulation/decrease, while green arrows indicate upregulation/increase by the compounds).

As the common molecular mechanism in NDs and neurotrauma involves the interplay of oxidative stress, neuroinflammation, and programmed cell death, a combination therapy has become utterly important. Henceforward, the bioactive compounds from the zombie fungus Cordyceps are the potential candidates in the treatment of such pathological conditions. They affect various pathways (Figure 2) involved in neuroprotection by alleviating ROS, reducing inflammation, restoring mitochondrial dysfunction, limiting apoptosis, and improving levels of antioxidant enzymes.

## 5. Safety and Toxicity

Cordyceps is a medicinal mushroom widely used as a health supplement. Like all other supplements, the US FDA has not approved it for safety concerns, but Cordyceps and its two dietary supplements have been approved by the Chinese National Medical Products Administration (NMPA) [230]. Cordyceps is relatively safe but may cause allergies, nausea, and stomach aches in some cases. Certain medications, such as antidiabetic, antithrombotic, and anticancer drugs [31,231,232], may interact with Cordyceps; hence, it is advised to consult a doctor before consuming them. The most prominent concern is the accumulation of heavy metals, especially arsenic, in naturally growing mushrooms in contaminated soils. The recommended dose of C. sinensis is 4 g/day for no more than 5 months [233]. Moreover, it is not recommended for patients with chronic kidney disease because it affects renal function [26]. As per the reports, cordycepin resulted in gastrointestinal and bone marrow toxicity in dogs [234,235], oosporein caused gout in avian species [234,236], and beauvericin might induce apoptosis and cell cytotoxicity [237]. Clinical trials have been conducted to investigate Cordyceps for immunomodulation [238,239,240,241], anticancer activity [242], respiratory function enhancement [243], insomnia management [244], prostate problem management [245], liver dysfunction alleviation [246], and increased tolerance to high-intensity exercise [32,247,248]. However, no clinical studies have investigated its neuroprotective activity.

## 6. Conclusions and Future Directions

Similarities in the pathophysiological mechanisms have been implicated in neurotrauma and neurodegenerative diseases. Both conditions are associated with oxidative stress, neuroinflammation, and glutamatergic excitotoxicity. The cascade of reactions can lead to apoptosis, necrosis, Ca^2+^ overload, protein aggregation, and mitochondrial dysfunction, affecting various signaling pathways (PI3K/AKT, TLR-4/NF-kB, Nrf/HO-1, ERK/p38/JNK] and eventually causing amnesia and brain damage.

Recent studies have demonstrated the role of dietary interventions in the control of oxidative stress, a key regulator of the pathogenesis of several diseases. Dietary components are known to boost mood, memory, and several other brain processes by maintaining a healthy oxidative state and improving synaptic function and neuronal plasticity. In this context, various studies validated the effectiveness of Cordyceps bioactives in protection against memory-related neuronal degeneration by antioxidant, anti-inflammatory, and anti-apoptotic properties. They exhibit anti-AChE activity, stimulate neurite outgrowth and neurotrophic growth factors, promote remyelination and dopamine release, and improve motor and cognitive function. These properties are important for maintaining synaptic plasticity and promoting neuron recovery. However, the pharmacodynamics of Cordyceps reveal a short half-life and low bioavailability, which limits therapeutic effect; hence, a system to increase bioavailability and enhance efficacy with curative practicality must be developed. Although Cordyceps contain several important compounds, only a few have been evaluated for their neuroprotective potential. Furthermore, no clinical studies have been conducted on the neuroprotective efficacy of the fungus. Thus, there is a need to investigate new bioactive compounds of Cordyceps to develop new drugs for preventing and treating neurodegeneration, in combination with preclinical and clinical safety assessments. Such studies will help in the design and development of safe and effective neuroprotective drugs.
